# Factors Influencing the Age at Discharge of Very Low Birth Weight Preterm Neonates From a Neonatal Intensive Care Unit in Eastern India: A Cohort Study

**DOI:** 10.7759/cureus.11889

**Published:** 2020-12-03

**Authors:** Jyoti R Behera, Gayatri Behera, Sanjay Kumar Sahu

**Affiliations:** 1 Pediatrics, Kalinga Institute of Medical Sciences, Bhubaneswar, IND; 2 Pathology, All India Institute of Medical Sciences, Bhubaneswar, IND

**Keywords:** neonatal intensive care unit, very low birth weight, preterm, age at discharge

## Abstract

Objective: To study factors that influence the age of discharge of very low birth weight (VLBW) preterm neonates from the Neonatal Intensive Care Unit (NICU).

Setting: This cohort study was conducted in the NICU, Department of Pediatrics, Kalinga Institute of Medical Sciences, Bhubaneswar, India, from April 2019 to December 2019.

Patients: Neonates whose birth weight was <1500 g and gestation <37 weeks were enrolled. Those having major congenital malformation were excluded.

Outcome: Age at discharge (in days).

Results: Out of 114 neonates enrolled, 84 neonates completed the study and discharged, 29 neonates died during the study, and one patient left against medical advice. Demographic, antenatal, neonatal factors, and mother’s participation in care of the baby were compared with respect to age at discharge in univariate analysis. Those found significant on univariate analysis were subjected to multivariate analysis. In the multivariate analysis model two independent predictors were observed, birth weight and gestation, which were inversely related to age at discharge.

Conclusion: Birth weight and gestation are the two most important factors which significantly influence the age at discharge from hospital of VLBW preterm neonates.

## Introduction

India has a large burden of very low birth weight (VLBW) babies, i.e. those who are <1500 g, account for only about 5% of all live births [1], but are responsible for most of the neonatal intensive care unit (NICU) bed occupancy and contributes to the most of the expenditure for neonatal care. VLBW babies in general have prolonged NICU stay by virtue of their specific set of problems like respiratory distress, apnea, intraventricular hemorrhage, need for mechanical ventilation, total parenteral nutrition, and time required for establishment of enteral feeding.

Provision of prolonged neonatal intensive care consumes both financial and medical resources and also increases the risk of nosocomial infections, disturbances in biological rhythm, poor parent-infant bonding, and possible failure to thrive after discharge. The current availability of NICU beds in India at present is far less than the required number. In order to meet the demands, there has been an increasing trend towards early discharge of neonates from NICUs.

A study from South Africa demonstrated that it was possible to discharge VLBW infants when they achieved weights of 1650 g or more [2]. There is, however, very limited data on the determinants of length of stay of VLBW neonates from under-resourced countries, including India.

## Materials and methods

The study was conducted in the NICU, Department of Pediatrics, Kalinga Institute of Medical Sciences, Bhubaneswar from April 2019 to December 2019. This was a cohort study that comprised a birth cohort of preterm neonates weighing <1500 g followed until discharge from the hospital. The neonates included in the study were inborn neonates who fulfilled the following criteria: birth weight <1500 g amd gestation <37 weeks. Amongst the neonates who fulfilled the above criteria, those having major congenital malformation were excluded. Primary outcome of the study was length of hospital stay (in days) as determined from the date of birth until the date of discharge. The study used a standardized discharge criteria for all neonates enrolled in the study. The newborn had to fulfill all the following criteria to be eligible for discharge from the hospital neonatal intensive care unit: 1) infant feeding completely from breast and/or spoon/cup, 2) weight gain of at least 15 g/day over three consecutive days, 3) clinically well (especially apnea free for three days), 4) maintaining normal body temperature on own, and 5) not on any injectable medication.

Since length of stay was likely to be influenced by birth weight of the newborn, the sample size was calculated to detect a difference in the mean length of stay between neonates with birth weight <1250 g and those with birth weight 1250-1499 g. In order to detect a difference of 14 days in the mean length of stay between neonates in the two weight categories with an absolute precision of five days with a confidence of 95%, it was calculated that 45 subjects needed to be enrolled. It was decided a priori that the study would enroll at least 50 VLBW preterm newborns.

Data collection

In all enrolled infants the following data were collected. All data were entered into a structured pretested performa. Data collection at enrollment were: 1) demographic data included maternal age, parental education, occupation, income and housing condition, 2) antenatal and intranatal data (these included mother’s parity, details of mother’s illness, details of obstetrics illness, details of mode of delivery, need for resuscitation at birth), and 3) neonatal data- at enrollment of neonate birth weight, gestational age (calculated from last menstrual cycle where first trimester dating by ultrasound was available or else by use of clinical scoring by Ballard score [3]), and whether an infant was a singleton or a product of multiple gestation was noted.

During the NICU stay, the infant was assessed every day and information with regard to baby survival, weight, temperature, and appearance of any illness was noted. The details of illness and the intervention carried out like supplemental oxygen therapy, mechanical ventilation, details of fluid therapy, intravenous medication like antibiotics, and surgical intervention done if any were recorded.

In each infant, the age of initiation at enteral feeding and age at attainment of full enteral volume of 150 ml/kg were noted. The sucking on the breast was noted each day and age at onset of effective sucking was recorded. Details of duration of nasogastric feeding, spoon-feeding, and breast feeding were also recorded. Availability of mother and her participation in care within the NICU was also recorded.

Statistical analysis

Data from a pre-coded data sheet were entered into Epi Info version 3.4.3 (2007) software. A univariate analysis was used to determine significant risk factors for length of stay. Those found significant on univariate analysis were analysed for independent prediction of length of hospital stay using a multivariate linear regression model. A subset analysis of risk factors for early and late discharge was also done. Continuous normally distributed data were analysed using student t-test and nonnormal data by the Mann-Whitney test. Proportions were compared by Chi-square or Fisher exact test. For all comparisons a probability of 5% was considered significant. 

Ethical approval

The institutional ethics committee approved the study protocol.

## Results

**Figure 1:**
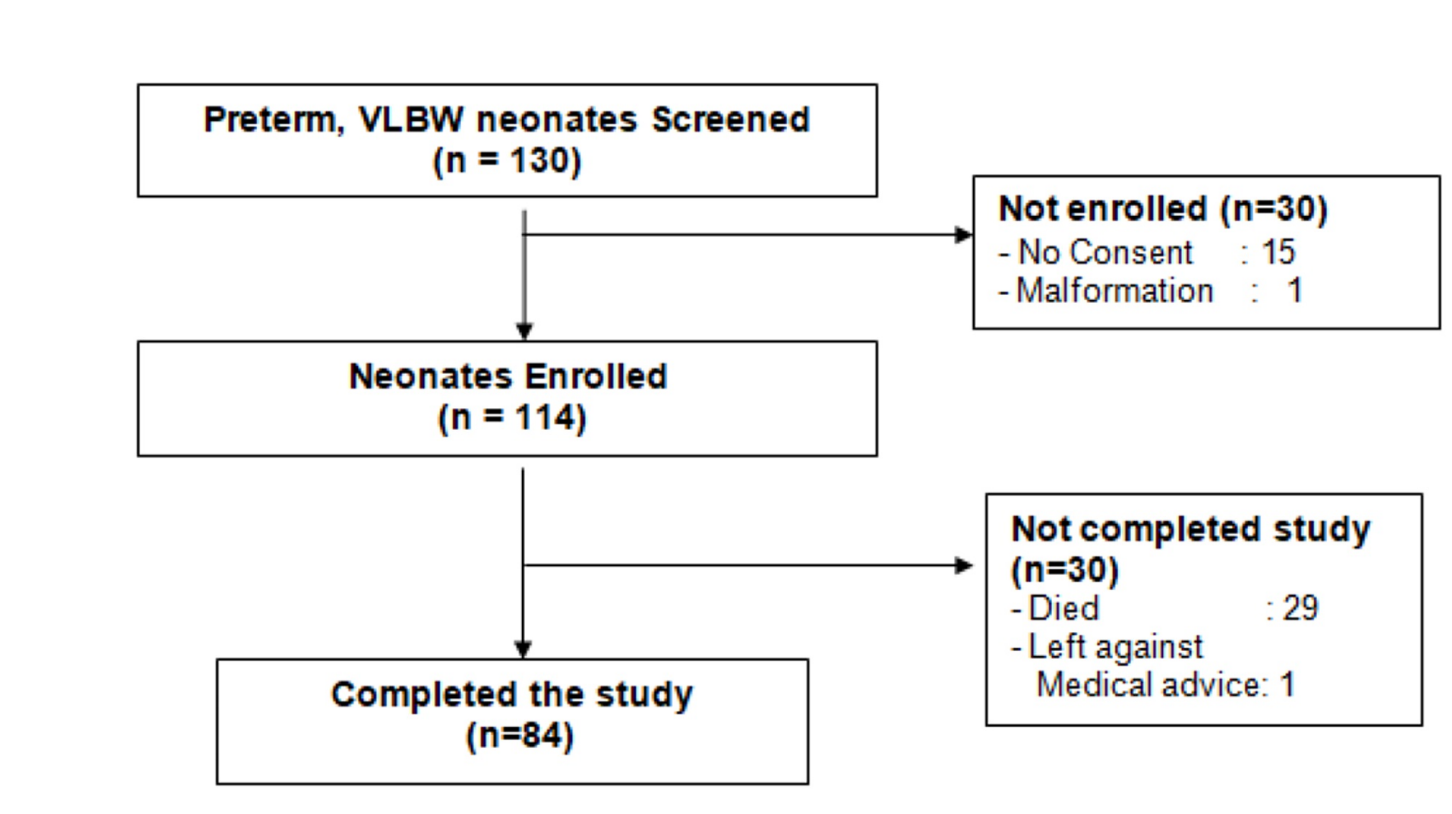
Flow of Study VLBW: very low birth weight

One hundred thirty preterm newborns weighing <1500 g were screened, of whom 114 were enrolled in the study. Reasons for non-enrollment are detailed in Figure 1. Of the 114 enrolled subjects, 84 were successfully discharged as per study criteria. Reasons for non-completion of the study amongst 30 subjects are detailed in Figure 1.

There were 84 (73.6%) newborns that completed the study. Table 1 provides a comparison of those who completed and who did not complete the study. The newborns in the group that did not complete the study were significantly shorter in gestation and smaller in birth weight. Median age of discharge for the entire group was 20 days (range seven to 58) and median gestation was 34 weeks (range of 30-36 weeks). In order to define “early discharge” for this cohort, the upper value of the lowest quartile was used as the cut-off, which corresponded to 14 days.

**Table 1:** Baseline characteristic comparison amongst groups completing and not completing the study

Variables	Completed Group (n=84)		Not completed Group (n=30)		P value
	Mean /Number(n)	SD(±)/ Percentage (%)	Mean/ Number	SD(±) /Percentage(%)	
Maternal age (yrs) Mean (sd)	25.8	±4.0	24.4	±3.2	0.8
Maternal education (n)					
Illiterate	15	17.9 %	11	36.7%	0.57
Primary school	13	15.5 %	5	16.6%	
Middle school	15	17.9 %	8	26.7%	
High school	21	25.0 %	5	16.6%	
Graduate	20	23.7 %	1	3.4%	
Per capita income (Rs)					0.19
< 750	50	59.5%	23	76.6%	
750-1499	24	28.6%	6	20.0%	
> 1499	10	11.9%	1	3.4%	
Birth weight (gm) mean (sd)	1321.6	±142.8	1135.5	±121.2	<0.001
Gestation (weeks) mean (sd)	33.4	±1.8	32.0	±1.6	<0.001

Early vs. late discharge

It was observed that infants in the early discharge group had significantly higher birth weight, higher gestation, had less resuscitation need, achieved enteral feed volume earlier, and transited to cup and breastfeeding earlier (Table 2). The late discharge group had significantly higher need for oxygen, mechanical ventilation, and intravenous medication. There was no significant difference with regard to etiology of illness.

**Table 2:** Early vs Late Discharge: Comparison of neonatal characteristics Apgar: Appearance, Pulse, Grimace, Activity, and Respiration, NICU: neonatal intensive care unit

Variables	Early discharge group (n=21)		Late discharge group (n=63)		P value
	Mean/Median	SD(±)/Range	Mean/Median	SD(±)/Range	
Birth weight(gm) Mean (sd)	1432.1	±53.7	1284.8	±144.4	<0.001
Gestation(weeks) Mean (sd)	34.8	± 1.3	32.9	± 1.8	<0.001
Apgar score mean (sd)					
-1 min	8.8	±0.4	7.9	±1.6	0.02
- 5 min	8.8	± 0.4	8.5	±0.9	0.04
Age enteral feed started (days) mean, (sd)	0	0	0.5	± 0.8	0.003
Age enteral feed volume of 150 ml/kg reached (days) (mean, sd)	3	±0.2	4.4	±1.9	0.001
Age transited to complete cup feeding (days) median (range)	7	4-11	15.5	0-48	<0.001
Age breast feed started (days) Median (range)	2	0-12	8	0-42	<0.001
Infant age when mother was involved in care for infant in NICU (day) Median ( range)	1	0-5	2	0-16	0.05

Almost all the mothers were available for care of their infants, except for two in the late discharge group who never participated in baby care because of illnesses (one had spinal tuberculosis and the other had hepatic encephalopathy). There was no significant difference between the groups with regard to the percentage of time mothers were available for infant care or the age at which mother began to get involved in care of her infant in the NICU.

Length of hospital stay (determinants)

Multiple linear regression was carried out using length of hospital stay (in days) as the dependant variables and all other variables as independent predictors. The two independent predictors observed were birth weight and gestation, which were inversely related to length of hospital stay (Table 3).

**Table 3:** Multivariate analysis of determinants of length of hospital stay Apgar: Appearance, Pulse, Grimace, Activity, and Respiration

Variables	Univariate		Multivariate		
	β coefficient	p value	β coefficient		p value
Birth weight	-0.04	< 0.001	-0 .028	< 0.001	
Gestation	-2.88	< 0.001	-1.54	0.005	
Maternal education	0.128	0.84	- 0.04	0.53	
Maternal age	2.91	0.11	- 0.102	0.9	
5 minute apgar	-2.78	0.03	- 0.82	0.44	
Neonatal illness	6.79	< 0.001	1.83	0.52	
Duration of oxygen therapy	7.55	<0.001	0.24	0.93	

## Discussion

VLBW preterm care is still a huge challenge in developing countries like India because of financial constraints and limited medical resources. Our study focuses on several maternal, social, and neonatal factors that are likely to determine the length of stay of VLBW preterm neonates in the NICU. Out of the factors studied, birth weight and gestation were found to be most significant which influence the timing of discharge of these babies. In a large population-based cohort, Altman et al. [4] observed that infants between 30-34 weeks with median gestation of around 33 weeks and median length of stay was around 3.5 weeks. These findings are very similar to those observed in the present study. In a study among late preterms (34-36 weeks of gestation), Pulver et al. [5] observed that the median length of stay of infants of 34 weeks was about 13 days. What was observed in the present study was that the median length of stay was about 17 days, if only babies with gestation of 34 weeks were considered.

In the Kotgal study [6], the mean length of stay for babies between 1250-1500 g was 33.3 days and for those between 1000-1250 g, it was on average of 49 days. One of the reasons behind longer hospital stay of infants in the Kotgal study when compared with similar weight strata in the present study was lesser gestation by about two weeks than subjects in the present study. Similarly in the study by Cassiro et al. [7], the median length of stay for infants <1500 g was of 56 days. In this study too the longer duration of stay was largely influenced by shorter gestation (32 weeks) as compared to subjects of the present study.

In a more recent study from under-resourced settings in Southern Africa, Mokhchane et al. [2] noted that mean length of stay was 25 days, which is significantly higher than the present study. Once again difference seems largely due to shorter gestation of infants of the study.

The study by Altman et al. [4] looked at determinants of length of hospital stay of 2,388 neonates born between 30-34 weeks across multiple hospitals in Sweden. Though found significant on univariate analysis, gestation was not found to be significant in multivariate analysis in contrast to observation in the present study where gestation found to be an independent risk factor. The lack of association of gestation with length of stay was probably because of the fact that neonatal morbidities overshadowed gestation and actually neonatal morbidities had served as proxy for gestational age. So this is logical considering that it is lower gestation which predisposes to illness and not vice-versa. Maternal age and multiple gestation were also found to be significant influencers in contrast to the present study. Mean maternal age was significantly higher (31.2 ±5.4) than what was observed in our study (25.8±4.0). There was no mother with >35 years age whereas the maternal age range extends to even 52 years in the Swedish study. Six hundred twelve multiple gestation babies were enrolled by Altman et al. whereas only four twins were in the current study. The significant difference in numbers might be the reason behind the contrasting findings of the two studies.

Pulver et al. [5] observed that in late preterm neonates significant factors that contribute to prolonged hospital stay were need for supplemental oxygen, phototherapy, intravenous medication, need for nasogastric feeding and assisted ventilation. Many of these observations were also proven to be significant in univariate analysis of the present study like neonatal illness and need for oxygen therapy.

Hintz et al. [8] had looked at predictors to time for discharge among extremely preterm infants (<27 weeks of gestation) and had observed that birth weight, gender, multiple births, ethnicity, need for ventilator support and need for resuscitation in the delivery room were important predictors. Even though in the present study, gestation of babies was >27 weeks, there are several similar risk factors like birth weight, maternal illness and need for resuscitation in the delivery room. However the present study did not note any difference in gender or ethnicity because of the small sample size and there were no ethnic groups.

Altman et al. [4] considered characteristics like availability of tertiary health care, fixed discharge criteria, small unit, and availability of domiciliary care in 21 hospitals in Sweden to influence length of stay in hospital of moderately preterm infants and found them to be significant. However, in the present study, though conducted in a tertiary care center with fixed discharge criteria, no domiciliary care was available, so we could not evaluate these characteristics.

## Conclusions

It may be concluded from the present study that birth weight and gestation have independent correlation with length of stay especially when minimum fixed criteria for early discharge using multiple parameters is utilized. Therefore units which handle infants with smaller gestation and smaller weight tend to have longer length of stay of these infants.

## References

[1] (2003). National Neonatal Perinatal Database Report 2002-2003. https://www.newbornwhocc.org/pdf/nnpd_report_2002-03.PDF.

[2] Mokhachane M, Saloojee H, Cooper PA (2006). Earlier discharge of very low birth weight infants from an under resourced African Hospital: a randomised controlled trial. Ann Trop Paediatr.

[3] Ballard JL, Khoury JC, Wedig K (1991). New Ballard Score, expanded to include extremely premature infants. J Pediatr.

[4] Altman M, Vanpee M, Cnattingius S, Norman M (2009). Moderately preterm infants and determinants of length of hospital stay. Arch Dis Child Fetal Neonatal Ed.

[5] Pulver LS, Denney JM, Silver RM, Young PC (2010). Morbidity and discharge timing of late preterm newborns. Clinic Pediatr.

[6] Kotagal UR, Perlstein PH, Gamblian V, Donovan EF, Atherton HD (1995). Description and evaluation of a program for the early discharge of infants from a neonatal intensive care unit. J Pediatr.

[7] Casiro OG, McKenzie ME, McFadyen L (1993). Earlier discharge with community-based intervention for low birth weight infants. Pediatrics.

[8] Hintz S, Bann CM., Ambalavanan N, Cotten CM, Das A, Higgins RD (2010). Predicting time to hospital discharge for extremely preterm infants. Pediatrics.

